# Opportunities and Challenges in the Genetics of COPD 2010: An International COPD Genetics Conference Report

**DOI:** 10.3109/15412555.2011.558864

**Published:** 2011-04-15

**Authors:** Edwin K Silverman, Jørgen Vestbo, Alvar Agusti, Wayne Anderson, Per S Bakke, Kathleen C Barnes, R Graham Barr, Eugene R Bleecker, H Marike Boezen, Kristin M Burkart, Bartolome R Celli, Michael H Cho, William OC Cookson, Thomas Croxton, Denise Daley, Dawn L DeMeo, Weiniu Gan, Judith Garcia-Aymerich, Ian P Hall, Nadia N Hansel, Craig P Hersh, Noor Kalsheker, James P Kiley, Woo Jin Kim, Diether Lambrechts, Sang-Do Lee, Augusto A Litonjua, David A Lomas, Stephanie J London, Masaharu Nishimura, Borge G Nørdestgaard, Christopher J O'Donnell, Dirkje S Postma, Milo A Puhan, Yohannes Tesfaigzi, Martin D Tobin, Claus Vogelmeier, Jemma B Wilk, Emiel Wouters, Robert P Young, Loems Ziegler-Heitbrock, William MacNee, James D Crapo

**Affiliations:** 1 Channing Laboratory (EKS, DLD, CPH, AAL) and Pulmonary and Critical Care Division (EKS, BRC, DLD, CPH, AAL), Brigham and Women's Hospital, Harvard Medical School, Boston, Massachusetts, USA; 2 University of Copenhagen /Hvidovre Hospital, Hvidovre, Denmark; 3 Institut del Torax Hospital Clinic, Universitat de Barcelona, and Centro de Investigacion Biomedica en Red de Enfermedades Respiratorias (CIBERES), Barcelona, Spain; 4 GlaxoSmithKline, Research Triangle Park, NC; 5 Institute of Internal Medicine, University of Bergen, Bergen, Norway; 6 Johns Hopkins Asthma & Allergy Center (KCB and NNH) and Department of Epidemiology (MAP), Johns Hopkins University, Baltimore, Maryland, USA; 7 Department of Medicine (RGB and KMB) and Departmen t of Epidemiology (RGB), Columbia University, Columbia University Medical Cen ter, New York, New York, USA; 8 Wake Forest University Health Sciences, Center for Genomics and Personalized Medicine Research, Winston-Salem, North Carolina, USA; 9 Department of Epidemiology (HMB) and Department of Pulmonology (DSP), University Medical Center Groningen, University of Groningen, Groningen, the Netherlands; 10 Division of Respiratory Sciences, Imperial College, London, United Kingdom; 11 Division of Lung Diseases, National Heart, Lung, and Blood Institute, Bethesda, Maryland, USA; 12 University of British Columbia, James Hogg iCAPTURE Center for Cardiovascular and Pulmonary Research, Vancouver, British Columbia, Canada; 13 Centre for Research in Environmental Epidemiology (CREAL), Barcelona, Spain; Municipal Institute of Medical Research (IMIM-Hospital del Mar), Barcelona, Spain; Department of Experimental and Health Sciences, Universitat Pompeu Fabra, Barcelona, Spain; CIBER Epidemiología y Salud Pública (CIBERESP), Barcelona, Spain; 14 Division of Therapeutics and Molecular Medicine (IPH) and Molecular Medical Sciences (NK), Nottingham Respiratory Biomedical Research Unit, University Hospital of Nottingham, Nottingham, United Kingdom; 15 Kangwon National University, Chuncheon, South Korea; 16 Vesalius Research Center, VIB and University ofLeuven, Leuven, Belgium; 17 Center for Chronic Obstructive Airway Disease, Division of Pulmonary and Critical Care Medicine, Asan Medical Center, Seoul, South Korea; 18 Department of Medicine, University of Cambridge, Cambridge Institute for Medical Research, Cambridge, United Kingdom; 19 Division of Intramural Research, National Institute of Environmental Health Sciences, National Institutes of Health, Department of Health and Human Services, Research Triangle Park, North Carolina, USA; 20 Hokkaido University School of Medicine, Sapporo, Japan; 21 University of Copenhagen andDept. of Clinical Biochemistry, Herlev Hospital, Copenhagen University Hospital, Herlev, Denmark; 22 Division of Intramural Research, National Heart, Lung and Blood Institute, National Institutes of Health, Bethesda, MD, and NHLBI's Framingham Heart Study, Framingham, Massachusetts, USA; 23 Lovelace Respiratory Research Institute, Albuquerque, New Mexico, USA; 24 Departments of Health Sciences & Genetics, University of Leicester, United Kingdom; 25 Division for Pulmonary Diseases, University of Marburg, Marburg, Germany; 26 Pulmonary Center, Boston University School of Medicine, Boston, Massachusetts, USA; 27 Departmentof Respiratory Diseases, University Hospital Maastricht, Az Maastricht, the Netherlands; 28 University of Auckland and Synergenz Bioscience Ltd, Auckland, New Zealand; 29 HelmholtzZentrum Muenchen, Germany; 30 University of Edinburgh, MRC Centre for Inflammation Research, The Queen's Medical Research Institute, Edinburgh, United Kingdom; 31 National Jewish Health, Denver, Colorado, USA

**Keywords:** COPD, Genetics, Association analysis, Consortium

## CONFERENCE PARTICIPANTS

Alvar Agusti, MD, PhD

Wayne Anderson, PhD

Per S. Bakke, PhD, MD

Kathleen C. Barnes, PhD

R. Graham Barr, MD, DrPH

Eugene R. Bleecker, MD

Kristin M. Burkart, MD, MSc

Bartolome R. Celli, MD

Michael H. Cho, MD

William O.C. Cookson, MD, DPhil

James D. Crapo, MD

Thomas Croxton, MD, PhD

Denise Daley, PhD

Dawn L. DeMeo, MD, MPH

Weiniu Gan, PhD

Judith Garcia-Aymerich, MD

Nadia N. Hansel, MD, MDPH

Craig Hersh, MD, MPH

Noor Kalsheker, MD

James P. Kiley, PhD

Woo Jin Kim, MD, PhD

Diether Lambrechts, MD

Sang-Do Lee, MD

Stephanie J. London, M.D., Dr.P.H.

William MacNee, MB, ChB, MD

Masaharu Nishimura, MD

Borge G. Nørdestgaard, MD, DMSc

Christopher O'Donnell, M.D.

Edwin K. Silverman, MD, PhD

Yohannes Tesfaigzi, PhD

Martin D. Tobin, MD

Jørgen Vestbo, MD, DMSc

Claus Vogelmeier, MD

John W Walsh

Jemma B. Wilk, DSc

Emiel Wouters, MD, PhD

Robert P. Young, MD, PhD

Loems Ziegler-Heitbrock, MD

REPORT AND RECOMMENDATIONS FROM CONFERENCE HELD JULY 13-14,2010, BOSTON, MASSACHUSETTS

## INTRODUCTION

Chronic obstructive pulmonary disease (COPD) is defined by the Global Initiative for Chronic Obstructive Lung Disease (GOLD) as a disease state characterized by airflow limitation that is not fully reversible (1). Cigarette smoking is the most important risk factor for the development of COPD. Although the dose-response relationship between cigarette smoking and pulmonary function is well-established, there is considerable variability in the reduction in FEV_1_ among smokers with similar smoking exposures (2, 3). The low percentage of variance in pulmonary function explained by smoking suggests that there could be genetic differences in susceptibility to the effects of cigarette smoking (4, 5). In addition to genetic factors, other environmental determinants such as indoor biomass smoke exposure can be important risk factors for COPD (6). A small percentage of COPD patients (estimated at 1-2%) inherit severe alpha-1 antitrypsin (AAT) deficiency, which proves that genetic factors can in-fluence COPD susceptibility. The discovery of AAT deficiency was a major factor in the development of the Protease-Antiprotease Hypothesis for COPD, which has been one of the prevailing models of disease pathogenesis for more than 40 years.

With the substantial impact of AAT deficiency on our understanding of COPD pathogenesis, it was natural to hope that the identification of other COPD susceptibility genes would lead to similar novel insights into COPD. Until recently, however, progress in the identification of additional genetic risk factors for COPD has been slow.

To facilitate the development of such research, a meeting of COPD genetics investigators was held on July 13-14,2010 in Boston. The goals of the meeting were:

1. To review the current state of COPD genetics research;
2. To discuss existing study populations for COPD genetics research throughout the world;
3. To consider opportunities for collaborations between different COPD research groups through an International COPD Genetics Consortium;
4. To recognize challenges in building COPD genetics collaborations and to discuss them openly; and,
5. To develop a framework for future collaborative studies.

### Current status of COPD genetics research

Many candidate gene association studies have been performed over the past 40 years, but the results have been largely inconsistent. These inconsistencies likely relate to a variety of methodological issues, including small sample sizes, variable definitions of case and control groups, failure to adjust for multiple statistical testing, and inadequate adjustments for population stratification and smoking exposure. Most of the studies describing COPD-associated polymorphisms were performed in White populations (7). A meta-analysis of 20 polymorphisms in 12 candidate genes involved in the protease-antiprotease balance and several an-tioxidant pathways showed that, after combining independent studies, many of these candidate genes had no association with COPD (8).

Another factor likely impeding the progress of identifying COPD susceptibility genes is the lack of accurate phenotypic characterization of this complex and heterogeneous disease. Airflow limitation determined by spirometry has been the most common approach to classify and monitor the disease. Structural changes of the lung including emphysema and small airway obstruction are the primary processes that affect lung function (9), but they are not easily discernable with the simple spirometric measures commonly used for phe-notyping COPD. Recent advances in characterizing pathologic changes such as emphysema and remodeling of the small and large airways by quantitative analyses of image data from multidetector computed tomography (CT), together with physiological testing, have been helpful to differentiate COPD phenotypes (emphysema-predominant, airway-predominant, or mixed)(10). Study populations that have chest CT data may help to better identify COPD-associated genetic variations (11). Other potentially relevant COPD phenotypes, such as cachexia and low exercise capacity, have not been widely analyzed in COPD genetic studies.

Perhaps the greatest problem in the candidate gene era of COPD genetic studies was improper candidate gene selection, which reflects our limited understanding of COPD pathogenesis. However, the application of genome-wide association studies (GWAS), which provide an unbiased and comprehensive search throughout the genome for common susceptibility loci, has changed the landscape of COPD genetics. Based on GWAS, three genetic loci have been unequivocally associated with COPD susceptibility, located on chromosome 4 near the *HHIP* gene, on chromosome 4 in the *FAM13A* gene, and on chromosome 15 in a block of genes which contains several components of the nicotinic acetyl-choline receptor as well as the *IREB2* gene.

In 2009, a series of studies provided convincing support for these three genetic loci in COPD susceptibility. Pillai and colleagues found genome-wide significant associations of the *CHRNA3/CHRNA5/IREB2* region to COPD (12). DeMeo and colleagues performed gene expression studies of normal vs. COPD lung tissues followed by genetic association analysis of COPD (13), suggesting that at least one of the key COPD genetic determinants in the chromosome 15 GWAS region was *IREB2.*

In the Framingham Heart Study (14), the *HHIP* region was associated with FEV_1_/FVC at genome-wide significance with replication of the effect on FEV_1_/FVC demonstrated in an independent sample drawn from the Family Heart Study, and this same region nearly reached genome-wide significance with COPD susceptibility in the Pillai paper (12). Recently, two papers published in Nature Genetics from large general population samples have provided strong support for the association of *HHIP* SNPs with FEV_1_/FVC (15, 16). One of these articles, from the CHARGE Consortium, also found evidence for association of FEV_1_/FVC with the *FAM13A* locus (15), which has been strongly associated with COPD susceptibility (17).

Moreover, several case-control studies from other European populations have replicated these findings by confirming significant associations to the chromosome 15q25 locus *(CHRNA3/CHRNA5/IREB2)* (18, 19), chromosome 4q31 locus *(HHIP)* (20, 21), and chromosome 4q22 locus *(FAM13 A)* (22). Thus, the frustration of inconsistent genetic association results in COPD from the beginning of the last decade has been replaced by optimism regarding the likely importance of the *IREB2/CHRNA3/CHRNA5, HHIP,* and *FAM13A* loci in COPD susceptibility.

### Advantages of creating large networks for genetic analysis

There are likely multiple additional COPD susceptibility genetic determinants that have not yet been identified. In many other complex diseases, the creation of large collaborative consortia has enabled highly powered genome-wide association studies that have led to the identification of multiple novel genetic susceptibility loci. For example, a Type 2 Diabetes mellitus consortium performed GWAS in 8,130 Cases and 38,987 Controls and identified multiple novel susceptibility loci (23). The International Lung Cancer Consortium found new SNPs that were associated with disease in Asian populations (24, 25). The ENGAGE consortium discovered sequence variants associated with smoking behavior within regions harboring nAChR genes *(CHRNB3-CHRNA6,* 8p11) and a nicotine-metabolizing enzyme (26). We anticipate that a similar collaborative consortium approach in COPD could lead to the identification of additional novel COPD genetic determinants.

### Gaps in current genetic knowledge

The most fundamental gap in current COPD genetics knowledge is that there are probably many genetic determinants of COPD, but only three genomic regions likely to contain such susceptibility loci have been conclusively identified. Moreover, the functional genetic variants within the three existing COPD GWAS regions remain to be found. To adequately analyze the various subtypes of COPD, studies that include multiple ethnic groups as well as multiple environmental factors that influence inflammation will be required in large sample sizes. More recently, some studies have combined results from several populations to increase the numbers of cases and controls. In more than 8300 subjects in seven study populations, the minor allele of a SNP in *MMP12* was associated with a positive effect on lung function and a reduced risk of COPD (27). The genome-wide association study that identified *FAM13A* included three sets of COPD cases and smoking controls (17). However, these studies are still underpowered to identify genetic determinants of small effect, and establishing a consortium of groups studying cigarette smokers may facilitate pooling large samples to identify genetic variants associated with COPD susceptibility.

## GENETIC TECHNOLOGIES AVAILABLE FOR AN INTERNATIONAL COPD GENETICS CONSORTIUM

It is desirable that the full power of modern genetic and genomic technology and techniques be brought to bear on COPD. Statistical genetic approaches should begin with meta-analyses of currently completed GWA studies, including imputation of polymorphisms from the 1000 Genomes Project. Analyses should routinely include epidemiologically important covariates such as sex, age at onset, and smoking history. Ancestry needs to be matched carefully between cases and controls, using, for example, principal component analyses. Multi-marker techniques to identify polygenic effects below the GWAS threshold maybe useful in identifying genes and pathways impacting on the disease.

Genome-wide SNP genotyping of several thousand or more cases is necessary, particularly using existing European panels of subjects that have not yet been genotyped and cases and controls of non-European ancestry. It is noted that there exists a wide range of previously genotyped European controls that could be used wherever possible.

Further meta-analysis of the full dataset should be completed after the additional genotyping. Ideally these results would be integrated with large-scale studies of other smoking-related diseases (particularly lung cancer and cardiovascular disease), with studies of smoking behavior and addiction, and studies of diseases characterized by compromised lung function (in particular, asthma).

Fine mapping of selected loci to identify functional variants will be necessary. This will include statistical approaches such as multiple regression as well as additional genotyp-ing. The particular importance of including ancestral groups of non-European origin in these analyses is noted, in order to use their differences in linkage disequilibrium patterns to break up linkage disequilibrium blocks, and to demonstrate generalizability of variants associated with COPD to the population at large.

Next-generation DNA sequencing approaches have the capacity to discover highly penetrant rare variants in common diseases such as COPD. Limiting sequencing to the ex-ome greatly reduces costs compared to whole genome sequencing approaches, while retaining much of the information that is likely to lead to the identification of disease-related rare variants. Although the value of exome sequencing has not yet been established in complex genetic diseases, it is desirable to explore the use of exome sequencing to search for rare mutations in patients with severe spectrum disease, including non-smokers with COPD as a separate group.

Genomic studies allow systematic investigation of pathways and networks of gene functions underlying disease (28). Investigations for COPD should include mapping of expression quantitative trait loci (eQTL) and network identification from measurements of global gene expression in airway biopsies and peripheral blood DNA samples. It would also be important to carry out eQTL mapping and network identification with global gene expression in current cigarette smokers and non-smokers. The investigation of methylQTL (using genome-wide methylation arrays) should similarly be implemented in order to explore epigenetic effects on COPD and related phenotypes.

Lastly, it is now possible to quantify bacterial colonization of airways using DNA and RNA sequencing techniques that address the hyper-variable bacterial 16S gene as well as metagenomic approaches that examine the global gene content and gene expression of human bacteria (29). It is therefore recommended that systematic studies of the microbiome be carried out in patients with COPD. These studies should include 16S sequencing for bacterial identification; metagenomic sequencing and measures of bacterial gene expression; and investigation of relationships of these measures to host gene expression and genotype.

## COPD PHENOTYPING AND KNOWLEDGE GAPS

### Clinical phenotypes

Precise definition and validation of clinical phenotypes are key prerequisites to identify the genetic basis of complex diseases, since a principal goal of genetic research is to identify specific genotypes that link to specific phenotypes (30, 31). From the genetics point of view, if current approaches in defining phenotypes are inadequate, the huge amount of currently available genotypic data cannot be optimally used (32). A recent consensus definition (11) proposes that a “COPD clinical phenotype” is “a single or combination of disease attributes that describe differences between individuals with COPD as they relate to clinically meaningful outcomes (symptoms, exacerbations, response to therapy, rate of disease progression or death).” Thus, for a COPD phenotype to be of use in a COPD genetics study, it has to be associated with clinically meaningful outcomes. Some inconsistent results published so far on the genetic basis of COPD may be due to the lack of an appropriate characterization of different clinical COPD phenotypes (intra-study variation), as well as to ethnic differences among studies (inter-study variation) (33).

The degree of airflow limitation remains the defining characteristic of COPD and thus its most important pheno-typic expression. However, there is sufficient evidence to support the need to consider additional phenotypic expressions in the characterization of patients with COPD. These include: 1) the degree, type and distribution of emphysema (discussed below); 2) the extent of airway wall thickening caused by inflammation; 3) the degree of hyperinflation expressed by the IC and the IC/TLC; 4) the presence of abnormal gas exchange (hypoxia and hypercapnia); 5) the presence of systemic involvement as measured by the BMI; 6) the exercise capacity whether measured in the laboratory (peak oxygen uptake) or in the field (6-minute walk test); and 7) the degree of functional dyspnea. These characteristics can practically be integrated into multidimensional tools such as the BODE index capable of providing a more comprehensive evaluation of COPD subjects (34). The determination of these phenotypic characteristics is not only scientifically interesting, but also clinically important because they confer prognostic value and more importantly, they determine response to therapy. Although COPD genetic studies have focused primarily on the presence/absence of COPD, analysis of these additional phenotypes could provide useful insights into COPD pathophysiology.

The study of COPD phenotypes is relevant to disease etiology, pathophysiology, and treatment. The identification of clinically relevant phenotypes would change the present view of COPD as a unique multicomponent disease (35, 36) to a syndrome with multiple phenotypic expressions, thus changing (and challenging) current taxonomy of chronic airway diseases (37). Regarding disease etiology, the identification of non-genetic determinants of diseases will also benefit from an appropriate definition of phenotypes. It is also likely that the traditional approach to address heterogeneity (i.e., stratification by socio-demographic, clinical, or environmental factors) is likely to lead to a reduction in statistical power (30). On the other hand, the identification of clinically relevant phenotypes should also lead to increased understanding of the underlying pathobiology that contributes to a particular phenotype (31). Despite the huge advances in our understanding of the pathology of COPD in recent decades, there have been few attempts to link COPD pathologies to clinical COPD phenotypes (38). Finally, it has been hypothesized that failure to identify COPD phenotypes may limit the power of therapeutic trials (39) as effective and safe therapy is likely to differ across phenotypes (31, 40). Several already existing examples illustrate this point, including the use of long-term oxygen therapy for COPD with chronic respiratory failure (but not for those with PaO2 values above 60 mmHg) (41, 42), the use of lung volume reduction surgery for patients with upper-lobe predominant emphysema and poor exercise capacity after rehabilitation (43), and, more recently, the development of roflumilast (a novel orally available anti-inflammatory drug) for only a subgroup of patients with COPD (those with chronic bronchitis) (44). Large, ongoing COPD studies may provide insight into phenotyping based on larger populations with more detailed descriptors.

### Chest CT phenotypes

The use of chest CT scans for determination of lung density was first described in the 1980s as a measure of the degree of emphysema in COPD (45). An important step was the introduction of digital image analysis software, such that the density of the entire lung can now be reported as the lower 15th percentile in Hounsfield units or the percentage of lung below a specific density mask threshold (e.g., < —950 HU) to define emphysema.

A more recent approach is the assessment of the thickness of the airwaywalls in order to determine the degree of airway remodelling. This was initially applied to asthma and more recently also to COPD (46). This approach appears to be robust for larger airways, and percent airway wall area can be used as a read-out (47). Although large airway dimensions correlate with small airway dimensions (48), direct assessment of the latter (airways <2 mm in diameter) is beyond the resolution of current CT scanning techniques. Quantitative assessment of chest CT scans for emphysema and airway disease provides an opportunity to define these two key phenotypes of COPD by objective criteria. There may also be further relevant CT-defined phenotypes that need more detailed study as to their clinical relevance and this includes emphysema distribution, emphysema pathological subtype (cen-trilobular vs. panlobular vs. paraseptal), the degree of mucus-mediated obstruction (plugging of airways), and bronchiec-tasis.

An important issue for multicenter trials is standardization of CT measurements across different clinical centers. Here the different brands and models of CT scanners, which use different scanning technologies, scanning protocols, and different algorithms for data processing, can affect the lung density and the airway wall results. Careful standardization including the use of phantoms for all scanners is required in order to be able to compare results. In spite of these problems, it maybe possible to obtain data on CT-assessed emphysema which could be compared in a multicenter study.

## EXISTING COPD STUDY POPULATIONS

At the Boston meeting, 38 study populations which included spirometric assessment of COPD and DNA sample collection were reviewed (Table 1). These studies included 20 case-control studies (or studies of cases only or controls only, which will all be included as “case-control” for this discussion), 16 population-based cohort studies (some of which had family components), and two family-based studies. Despite the smaller number of studies, a much larger number of total subjects (> 130,000) were available in the population-based cohort studies than in the case-control studies (approximately 38,000). The majority of studies have been performed in White populations. Most of the case-control studies include post-bronchodilator spirometry and a minimum number of pack-years of smoking criterion for inclusion, while most of the population-based cohort studies do not (Table 2). A surprisingly large fraction of case-control studies as well as some of the population-based studies included chest CT scan assessment. COPD exacerbations were also assessed in many studies.

**Table 1:** International COPD Genetics Consortium study populations—Genetics

		Current sample size*			Genome-wide SNP genotyping availability							
Study name	Study type	All COPD	Severe COPD	Control	All COPD	Severe COPD	Control	Estimated final sample size for genetic studies	Genome-wide SNP genotyping platform	Country	Race/ethnicity	Ref
ANOLD**	Case-Only	1011	431	0	0	0	0	1,200	None	Nine asian countries	All Asian	
COPACETIC	Case-Control	1029	46	1958	1029	46	1958	15,000	Illumina 610	Europe	AllWhite	(56)
COPDGene**	Case-Control	3590	1791	4131	2481	1189	3014	10,000	Illumina omni-express	USA	2/3 non-Hispanic Whites (NHW) and 1/3 African Americans (AA)	(57)
COSYCONET**	Case-Control	3000	1500	>5000	0	0	0	>6000	Not determined	Germany	AllWhite	
ECLIPSE	Case-Control	1839	1003	196	1839	1003	196	2043	Illumina 550	Europe, N. America, Australia	AllWhite	(58)
EU COPD/East midlands consortium	Case-Control	1616	1050	912	0	0	0	2528	Not determined	Europe	AllWhite	(59)
EvA**	Case-Control	290	78	230	0	0	0	1,200	Illumina	Europe	AllWhite	
GenKOLS	Case-Control	933	386	919	933	386	919	1853	Illumina 550	Norway	AllWhite	(12)
Hokkaido	Case-Control	274	45	150	0	0	0	500	None	Japan	All Asian	(60)
ICE COLD	Case-Only	411	142	0	0	0	0	411	None	Switzerland/Netherlands	AllWhite	(61)
KOLD**	Case-Control	321	134	305	0	0	0	700 COPD Cases	None	Korea	All Asian	(62)
Leuven	Case-Control	800	400	300	0	0	0	1,250	Not applicable	Belgium	AllWhite	(19)
Lung Health Study	Case-Only	5887	0	0	4126	0	0	4191	Illumina 660W	USA and Canada	4126 Whites, 65 AA	(63)
New Zealand	Case-Control	458	277	488	0	0	0	946	Not applicable	New Zealand	AllWhite	(18)
NETT	Case-Only	366	366	0	366	366	0	366	Illumina 610	USA	AllWhite	(43)
PAC COPD	Case-Only	342	159	0	0	0	0	342	0	Spain	AllWhite	(64)
SPIROMICS**	Case-Control	0	0	0	0	0	0	3200	Not Determined	USA	Not Determined	-
Transcontinental: Korea**	Case-Control	80	80	179	0	0	0	600	None	Korea	All Asian	-
Transcontinental: Poland	Case-Control	302	302	333	0	0	0	635	None	Poland	AllWhite	-
Wake Forest	Case-Control	490	192	509	490 (in 2011)	192 (in 2011)	509 (in 2011)	1,000	Illumina	USA	91% White 8% AA	(65)
Atherosclerosis Risk in Communities (ARIC)+,#	Pop-Based	1194	586	8337	943	481	6602	7545	Affymetrix 6.0 + IBC chip (Both Whites and African Americans)	USA	White	(15)
		247	155	3315	185	30	1607	1792			African American	
Cardiovascular Health Study (CHS)+,#	Pop-Based	402	292	2183	402	292	2183	2585	Illumina 370CNV beadchip + IBC chip (Both Whites and African Americans)	USA	White+	(15)
		123	31	277	123	31	277	400			African American#	
Cleveland Family Study#	Family/Pop-Based	17	4	203	0	0	0	220	IBC chip	USA	White#	
		29	4	159	29	4	159	188	IBC chip + Affymetrix 6.0		African American#	
Copenhagen City Heart	Pop-Based	∼2000	n/a	∼8000	0	0	0	∼10,000	None	Denmark	n/a	
Copenhagen General Population	Pop-Based	∼6000	n/a	∼54,000	0	0	0	∼60,000	None	Denmark	n/a	
Coronary Artery Risk Development in Young Adults (CARDIA)#	Pop-Based	31	2	1148	0	0	0	1179	IBC chip	USA	White#	
		25	2	969	25	2	969	994	IBC chip + Affymetrix 6.0		African American#	
EMCAP	Pop-Based	200	n/a	450	0	0	0	657	None	USA	70% White, 10% AA, 10% Hispanic, 10% Asian	(66)
Framingham Heart Study+#	Family/Pop-Based	571	274	5866	571	274	5866	6437	Affymetrix 500 + 50K gene-centric chip	USA	AllWhite#	(14)
Jackson Heart Study#	Pop-Based	182	36	1982	182	36	1982	2164	Affymetrix 6.0 + IBC chip	USA	African American#	
Lifelines	Family/Pop-Based	592	10	2506	592	10	2506	20,000	Illumina Human Cyto SNP-12 V2	Netherlands	MostlyWhite	(67)
Lovelace Smokers Cohort	Pop-Based	500	100	1700	0	0	0	2900	None	USA	20% Hisp 80% NHW	(68)
Multi-Ethnic Study of Atherosclerosis (MESA) lung study+,#	Pop-Based	384	53	2651	∼350	∼50	∼2500	3965	Affymetrix 6.0 + IBC chip	USA	35% NHW*,# 25% AA# 25% Hisp# 15% Asian#	(69)
MESA Lung SHARe	Family/Pop-Based	‡	‡	‡	‡	‡	‡	4200	Affymetrix 6.0	USA	30% NHW#,* 30% AA# 30% Hisp# 10% Asian#	
Normative Aging Study	Pop-Based	191	n/a	1024	0	0	472	1215	Illumina 610	USA	AllWhite	(17, 27)
Rotterdam Study (RS1, RS2 and RS3 combined)+	Pop-Based	209	136	2672	209	136	2672	2881	Illumina Infinium II 550K	Netherlands	AllWhite	(15)
SpiroMeta++	Pop-Based	3278	n/a	17,523	356	n/a	3,730	63,079	Multiple GWAS platforms (n = 24, 756); multiple non-GWAS platforms (n = 38, 314)	Europe and Australia	AllWhite	(16)
Vlagtwedde/Vlaardingen	Pop-Based	351	27	991	0	0	0	1342	None	Netherlands	AllWhite	(70)
		Probands	Relatives		Probands	Relatives						
Boston Early-Onset COPD	Family	200	∼1000		0	0		1200	None	USA	98%White	4
International COPD Genetics Network	Family	983	1876		0	0		2859	None	North America and Europe	Mostly White	(71)

* Note that “All COPD” and “Control” were defined differently within each study and/or consortium. “Severe COPD” was defined as GOLD 3-4 using post-bronchodilator spirometry if available, and pre-bronchodilator spirometry if not available. For the CHARGE consortium studies, severe COPD was defined as noted below.

** Indicates that study population is still being recruited.

+ Participating in the *Cohorts for Heart and Aging Research in Genomic Epidemiology* (CHARGE) consortium which includes onlyWhites from these cohorts. In CHARGE, COPD was defined as FEV_1_<LLN and FEV_1_/FVC<LLN. Severe COPD was defined as FEV_1_/FVC < LLN and FEV_1_ < 65% Predicted. Controls were defined by FEV_1_, FEV_1_/FVC and FVC all > LLN.

# Participating in the Candidate Gene Association Resource (CARe) consortium (72). For African Americans in CARe, “COPD” was defined as pre-BD ratio <0.70 and FEV1%pred <80%; “severe COPD” as pre-BD ratio <0.70 and FEV1%pred <50%; and controls as pre-BD ratio >0.70 and FEV1%pred >80%. Note that for cohorts participating in both CHARGE and CARe, definitions of COPD, severe COPD, and control status used in this table differ for Caucasians and African-Americans from the same participating cohort. Additionally, for the ARIC cohort, case and control counts for the total (“current”) sample of African-Americans are based on the CHARGE definitions rather than the CARe definitions.

++ SpiroMeta-Cases: FEV_1_ <80% predicted and FEV_1_/FVC <0.7; Controls FEV_1_>80% predicted and FEV_1_/FVC ratio of >0.7. Numbers of individuals known to be cases and known to be controls are shown (further data collection is underway; the number of known COPD cases and documented controls will increase when this information is received).

‡ Characterized with CT scan only.

**Table 2:** International COPD Genetics Consortium study populations—Phenotypes

				Spirometry		Chest CT			Other Phenotypes		
Study	Age Range	Gender (% Male)	Minimum Pack-Years	Pre-BD	Post-BD	Obtained?	Visual Assessment	Quantitative Assessment	Exac**	Co-Mor**	Lung Function Decline
ANOLD	40-91	94	10	Yes	Yes	No	-	-	Yes	Yes	No
COPACETIC	50-80	98	20	Yes	Some	Yes	No	Yes	Yes	No	Yes
COPDGene	45-80	52	10	Yes	Yes	Yes	No	Yes	Yes	Yes	No
COSYCONET	>40	n/a	0	Yes	Yes	Some	Yes	Yes	Yes	Yes	Yes
ECLIPSE	40-75	66	10	Yes	Yes	Yes	Yes	Yes	Yes	Yes	Yes
EU COPD/East Midlands Consortium	Mean 66	70	10	Yes	Yes	No	-	-	No	Yes	Some
EvA	45-75	65	5	Yes	Yes	Yes	No	Yes	No	Yes	No
GenKOLS	40-81	66	2.5	Yes	Yes	Yes	Yes	Yes	Yes	Yes	Some
Hokkaido	41-87	94	12	Yes	Yes	Yes	Yes	Yes	Yes	Yes	Yes
ICE COLD	Mean 66	57	0	No	Yes	No	-	-	Yes	Yes	Yes
KOLD	45-82	92	10	Yes	Yes	Yes	Yes	Yes	Yes	Yes	Yes
Leuven	43-90	75	15	Yes	Yes	Yes	Yes	No	Yes	Yes	No
Lung Health Study	35-60	63	10	Yes	Yes	No	-	-	Yes	Some	Yes
New Zealand	40-85	60	15	Yes	No	No	-	-	No	Yes	No
NETT	Mean 67	73	0	Yes	Yes	Yes	Yes	Yes	Yes	Yes	No
PAC COPD	Mean 68; Range 44-86	93	0	Yes	Yes	Yes	Yes	Yes	Yes	Yes	Yes
SPIROMICS	TBD	TBD	TBD	Yes	Yes	Yes	Yes	Yes	Yes	Yes	Yes
Transcontinental: Korea	40-80	97	10	Yes	Yes	No			Yes	Yes	No
Transcontinental: Poland	40-80	69	10	Yes	Yes	No			Yes	Yes	No
Wake Forest	42-88	96	20	Yes	No	No	-	-	No	Yes	Yes
ARIC	45-64	45	0	Yes	No	No	-	-	Yes	Yes	Yes
Cardiovascular Health Study	65-95	43	0	Yes	No	No			No	Yes	Yes
Cleveland Family Study	23-88	48	0	Yes	No	No	-	-	No	Yes	No
Copenhagen City Heart	n/a	n/a	0	Yes	n/a	No	-	-	Yes	Yes	n/a
Copenhagen General Population	n/a	n/a	0	Yes	n/a	No	-	-	Yes	Yes	?
CARDIA	27-44	45	0	Yes	No	No	-	-	No	Yes	Yes
EMCAP	>60	50	10	Yes	Some	Yes	Yes	Yes	Yes	Yes	Yes
Framingham	19-92	46	0	Yes	Some	In Subset	Pending	Pending	No	Yes	Yes
Jackson Heart Study	21-93	38	0	Yes	No	No	-	-	No	Yes	No
Lifelines	21-88	41	0	Yes	No	No	-	-	No	Yes	No
Lovelace Smokers Cohort	40-75	22	15	Yes	Yes	No	-	-	Yes	Yes	Yes
MESA Lung	45-84	49	0	Yes	sPe	Yes	Pending	Yes	Yes	Yes	Pending
MESA Lung SHARe	45-84	50	0	No	No	Yes (cardiac)	No	Yes	No	Yes	No
Normative Aging Study	21->80	100	0	Yes	No	No	-	-	No	Yes	Yes
Rotterdam Study	>45	45	0	Yes	No	No	-	-	No	Yes	Pending (RS1)
SpiroMeta	8-93*	42*	0	Yes	In a subset	No	-	-	No	No	No
Vlagtwedde/Vlaardingen	35-79	51	0	Yes	No	No	-	-	No	Some	Yes
Boston Early-Onset COPD	>10	42	0	Yes	Yes	No	-	-	Yes	Yes	No
International COPD Genetics Network	45-65	46	5	Yes	Yes	In Subset	Yes	Yes	Yes	Yes	No

* For a subset of individuals (n = 20, 288) with information on age and gender currently available.

** Abbreviations: “Exac“ = Data available regarding COPD exacerbations; “CoMor“ = Data available regarding co-morbid diseases.

Using the reported definitions of COPD and non-COPD from each study, there are approximately 39,600 COPD cases and 131,600 control subjects in the combined set of case-control and population cohort studies (Table 3). In these case-control and population cohort studies, there are approximately 14,700 cases and 37,600 controls reported to have genome-wide SNP genotyping currently available.

**Table 3:** International COPD Genetics Consortium: Approximate current study population sample sizes

	Cases	Controls
Case-Control Studies (or Case-Only Studies)	23,039	15,610
Cohort Studies	16,526	115,956
Combined Set	39,565	131,566
GWAS in Combined Set Currently Available	14,741	37,612

Notes: For this table, approximate sample sizes from Table 1 were used as actual values. Only subjects with spirometry from Table 1 were included in these calculations (MESA Lung SHARe was excluded). Population-based studies with a family component (e.g., Framingham) are listed under “Cohort Studies,” while other pedigree-based studies (e.g. International COPD Genetics Network) have been excluded. For the “GWAS in Combined Set Currently Available,” only subjects with available genotyping by December 2010 were included.

## WORKSHOP RECOMMENDATIONS FOR CREATION OF AN INTERNATIONAL COPD GENETICS CONSORTIUM

### Rationale and vision

At the Boston meeting, the participants identified multiple advantages of creating a COPD genetics research consortium, and strongly endorsed this approach. Larger sample sizes of cases and controls will definitely increase power to detect COPD susceptibility loci. The potential to assemble large numbers of severe COPD subjects that are clearly affected is a major advantage, since relatively small numbers of severely affected subjects have been included in most individual studies. Similarly, the opportunity to perform pooled analyses of chest CT phenotypes was seen as a major strength, as long as the technical challenges of different CT scanning and analytical protocols can be overcome. Opportunities to study other COPD-related phenotypes, including COPD exacerbation frequency, lung function decline, and lung cancer, were also recognized. Although many of the participating studies do not yet have genome-wide SNP genotyping, these studies provide opportunities to replicate initial GWAS findings in large numbers of additional subjects. In addition to studies of main genetic effects, a large COPD genetics consortium would improve the statistical power to study gene-by-environment interactions.

Although the studies listed above could be performed in a fairly short time-frame, potential future advantages of a COPD genetics consortium were also appreciated. Such a consortium could provide a framework for future genetic collaborations in exome sequencing and whole genome sequencing, as well as in other genetic/genomic areas (e.g., epi-genetics, gene expression). There would likely be increased standardization of study protocols and procedures for future studies (e.g., imaging, questionnaires) and the potential for collaborative studies of non-genetic issues (e.g., phenotypes, biomarkers). Limiting duplication of research effort could likely be accomplished as well. Overall, there was general consensus that a COPD genetics consortium would have a high likelihood of significantly advancing knowledge in the field.

In addition to these advantages of forming a collaborative consortium, a variety of challenges were identified. It was recognized that there are academic realities including the need for individual research groups to demonstrate academic productivity to renew funding and promote research personnel. Some COPD genetics collaborations already exist, and a goal was not to interfere with those existing relationships. Although studies that include reasonable numbers of COPD cases and control subjects could be analyzed individually and combined using meta-analytical approaches, the optimal approach for utilizing studies of COPD cases only or controls only was not as clear.

A variety of challenges related to phenotypic characterization were also identified. Substantial variation exists in the definitions of cases and controls between studies (e.g., physiologic measurements of lung function using GOLD criteria or use of lower limit of normal [LLN]), as well as in spirometry protocol (e.g., pre- vs. post-bronchodilator). Some pheno-types (e.g., imaging) maybe difficult to combine across studies due to technical issues. There are important variations in study populations (e.g., race/ethnicity, smoking history, exclusion of subjects with other illnesses, other criteria used for selection, study design, and informed consent restrictions) and genetic analysis approaches (e.g., variation in genotyping platform, data cleaning, analytical approaches, and data sharing).

Despite these challenges, there was general agreement that the advantages of collaboration far outweighed the limitations, and that a transparent and open collaboration could overcome most of the challenges. To be successful, the needs and rights of each contributing study will need to be respected. Based on the enthusiastic support for an international COPD genetics consortium from the Boston meeting participants, the research projects amenable to this consortium approach and an organizational structure for the consortium were discussed.

### Feasibility of collaborative COPD genetics studies

Although the development of large consortia of thousands of subjects may obviate some of the issues that have contributed to non-replication of previous COPD genetic studies (such as power limitations germane to smaller studies), the inclusion of data from a large number of studies presents unique challenges and opportunities.

### Smoking exposure and penetrance

Despite the challenges of disease gene discovery in complex disease, there are some striking advantages to studying the genetics of COPD (COPD strictly being a syndrome not a specific disease). First, one of the most important features of studying the genetics of COPD is that the key environmental exposure of cigarette smoking is known and quantifiable in the setting of a gene-by-environment interaction. In contrast to most other complex diseases, the majority of COPD can be attributed to a single exposure (cigarette smoking) which can be crudely quantified, by intensity (cigarettes/day) and/or total exposure (pack-years), across both cases and controls in geographically diverse populations. The central role of smoking exposure in genetic susceptibility is illustrated by the divergent outcomes in people with alpha-1 antitrypsin deficiency based on their smoking history (49).

Second, although COPD is a syndrome encompassing both emphysema and small airway disease that are present in varying degrees, both are characterized by irreversible airflow limitation (reduced FEV_1_ and FEV_1_/FVC ratio), which can be measured by simple spirometry in population studies. From an epidemiological perspective, FEV_1_ (after age, gender, race, and height adjustment) provides a good starting place from which to define COPD, as it is a highly heritable trait (50) regardless of the heterogeneity of COPD subjects. Moreover, with increasing smoking exposure, FEV_1_ defines susceptible and resistant smokers with an increasingly bimodal distribution supportive of a genetic basis (2, 51-53) and possibly a threshold effect. Comparing smokers at either end of the FEV_1_ spectrum but with comparable smoking exposure, so called “extreme phenotypes” (54), may help to overcome minor differences in spirometric criteria defining the COPD phenotype.

Despite these characteristics of COPD as a complex genetic disease, there remain significant challenges in combining population-based and case-control samples. Many studies that can be included in a collaborative COPD meta-analysis have not taken detailed smoking histories or validated intensity of current smoking via measurements of coti-nine levels. Although reporting bias is a concern, self-report of cigarette smoking has been demonstrated as a reliable assessment.

Some of the studies proposed for inclusion in this consortium have focused on a minimum amount of smoking exposure for enrollment, while others have not. Similarly, some studies have focused on the heavy smoker, and some have included a range of exposures. Including all studies allows for a reasonable attempt to achieve the necessary power to assess genetic main effects, but also gene-by-smoking interactions through stratification and/or adjustment. In addition, genetic insights into COPD will be gleaned by not only studying those genes that associate with COPD susceptibility, but also genes that may portend protective resistance to COPD in subjects with an extremely high number of pack-years but normal lung function.

### Heterogeneity of COPD

First and foremost in the planning and organization of large consortia with the goal of meta-analysis, the heterogeneity of phenotypes across studies needs to be addressed. This is an issue by no means limited to respiratory genetic studies. A paramount challenge in studies of COPD has been the inherent heterogeneity of the disease, variable effects of smoking exposure on penetrance (described previously), and the importance of defining disease subtypes. In addition, not all studies have performed pre- and post-bronchodilator spirometry, and many studies have not been sufficiently resourced to undertake chest CT scanning to phenotype COPD pathologically. Even in the presence of post-bronchodilator spirometry, issues of spirometric diagnosis of COPD based on using GOLD criteria versus lower limit of normal may contribute to phenotypic heterogeneity. This is likely to be minimized in those case-control studies comparing more extreme (susceptible vs resistant) phenotypes where misclassi-fication of cases or controls based on variation in spirometry-based definition is likely to be minor. However, severity of COPD is an issue that needs careful consideration given the strong association between aging and loss of lung function. Moreover, it is noted that a spirometric diagnosis of a resistant smoker does not obviate some misclassification as smoking-related emphysema may be present despite normal spirometric measures.

A major strength of this consortium will be the availability of data from both spirometry and CT scans of the lungs for parsing subjects by emphysema status (and also within COPD cases for emphysema versus airway predominant disease). Although CT scanning presents challenges (lack of uniformity of scanner technical characteristics, scanning protocols, radiation dosing, scoring of emphysema severity/distribution, etc.), computerized approaches to process and analyze chest CT scans may be useful in harmonizing CT scan data, and will likely assist in refinement of COPD subtypes.

### Ethnic heterogeneity/population substructure

Although the inclusion of data from Caucasian, African, Native American, and Asian subjects may lead to false positive and/or false negative association findings due to population substructure, contribution of all subjects’ data to the power of the overall analysis, and to the race-specific genetic association analyses of COPD, are major strengths of performing a multi-ethnic consortium. There are many existing examples of disease associations confined to specific ethnic groups. Primary analyses would be conducted within each ethnic group, followed by comparison of association results between groups.

### Study-specific issues

Although the inclusion of many studies of COPD may increase the power due to an increase in total subject number, presently a minority of COPD studies have GWA data. Given the large burden of disease accounted for by COPD, this void of GWA data in and of itself supports the importance of efforts to accrue more GWA data. The consortium will include GWA data on both population-based cohorts and case-control studies. In the population-based cohorts, the contribution of genetic variants on lung function can be explored in settings other than chronic smoking.

There is a need for more GWA data in case-control studies, where smoking has been accounted for and other important exposures have been examined. Case-control studies can also allow investigation of the role in disease of variants shown to be associated with lung function in population-based studies. The case-only studies can be added to the case-control studies in a mega-analysis of individual genotype and phenotype data, or in circumstances where data can be combined or compared with the controls derived from other studies. The consortium is also fortunate enough to have large prospective studies in which the genetic determinants of rate of decline in lung function (or other aspects of disease progression) can be studied; this could be insightful, for example, for those variants shown to be associated with COPD or cross-sectional lung function measures.

Other study-specific issues include variations in enrollment criteria, age ranges of subjects, and rates of co-morbid conditions including obesity which may affect lung function. Of these, variations in smoking history (described above) and current smoking status are potentially the most essential, as some of the COPD studies had minimum amounts of smoking exposure required for eligibility, and include a mix of current and former smokers. Given that gene-by-smoking interactions are crucial to include in genetic analyses (2, 51-53) this variable inclusion of subjects may seem to be a limitation.

For genetic studies there is likely enrichment in genetic effects in those subjects who develop COPD at a very young age as well as those smokers who remain resistant to both COPD and emphysema at very old ages. Associations will need to be re-examined with stratification by age of disease onset, total pack-year exposure and current smoking status where the data are known.

Variable rates of comorbidities in the different COPD studies may impact genetic associations with lung function (such as the association of diabetes with lower lung function) but the inclusion in genetic analysis of the most diverse group with COPD may increase the likelihood that positive associations are true positive findings.

### GWAS platforms and data cleaning

As has been the challenge in other complex diseases such as diabetes, the platforms used for genome-wide genotyping have varied. There is variable inclusion of SNPs leading to differential coverage of genes on a given platform. However, this reality has led to the availability of imputation methods to overcome the differences between GWA arrays; these novel *in silico* tools allow for the development of a larger study population less constrained by choice of genotyping technology. In addition to the genotyping platform, approaches to data cleaning may vary between bioinformatics groups. However, a harmonized approach to data cleaning is mandatory.

### Sharing of individual subject data

The protection of human data and subject privacy is paramount and the ability to share individual level genotype results maybe limited. Thus, the performance of “mega-analysis” in which individual-level genotype and phenotype data would be shared, though remaining a worthwhile eventual goal, was judged not to be essential to progress at this time. However, meta-analytic approaches that use study-wide association data (p-values) weighted by study size or by inverse variance have been shown to be as powerful as mega-analysis approaches that utilize subject level data (55). Thus for identification of common variants for COPD (at least 5% minor allele frequency) meta-analytic approaches will provide important insights into COPD.

### Plan for initial meta-analysis

A preliminary design for the initial collaborative genetic association meta-analysis for the consortium was created at the Boston meeting. Two key genome-wide association analyses were proposed: 1) All COPD vs. Controls, and 2) Severe COPD vs. Controls. The precise definitions of All COPD and Severe COPD remain to be determined. Within each study population that has existing genome-wide SNP geno-typing data, standard quality control approaches will be used to clean the data, including criteria for exclusion of SNPs with low call rates, low minor allele frequency, departures from Hardy-Weinberg equilibrium, and differential rates of missing data between cases and controls, and exclusion of individuals with low call rates or exhibiting cryptic relatedness among unrelated samples.

Standard approaches to genotype imputation will be applied in each study, followed by a similar approach to population stratification adjustment within each study using adjustment with principal components for genetic ancestry. Genome-wide association analysis for the two COPD affection status phenotypes (all COPD and severe COPD) will be performed within each study, with separate analyses in subjects of Caucasian, Asian, and African ancestry. Meta-analysis of GWAS will be performed within each major racial group using inverse variance weighted meta-analysis methods to account for differences in sample size and imputation quality across genotyping platforms, followed by comparison of association evidence between major racial groups. Finally, replication genotyping and association analysis of the most interesting SNPs will be performed in the remaining study populations without genome-wide SNP data.

### Structure of the Consortium

The mandate of the International COPD Genetics Consortium is to find common and rare genetic determinants of COPD; to identify COPD subtypes and their genetic basis; and to use this information to develop new disease classifications and therapeutic interventions. Based on the discussions at the Boston meeting, it was recommended that research studies including COPD and control subjects would be invited to participate if they collected high quality spirom-etry data and DNA samples, and if the study met a minimum sample size. The expectation is that the studies will include at least 200 COPD cases and 200 controls, but review of specific studies is possible if those criteria are not met. For studies that include case-only collections, they would be encouraged to find appropriate sets of control subjects for genetic association analysis; if not available, those COPD study populations could be included in studies of COPD progression or CT subtypes. Study populations meeting these criteria that were not represented at the Boston meeting will be welcome to join this international collaborative effort.

Several committees will be created to perform the consortium research and administration, including a Steering Committee (in charge of major decisions); Planning/Executive Committee (routine operations); Phenotype Harmonization; Imaging Committee; Genotyping and Genomics Core; and Analysis Core.

The International COPD Genetics Consortium has the potential to provide short-term results by providing highly powered genome-wide association studies of COPD susceptibility, and long-term results by facilitating the study of other COPD-related phenotypes and other genomic outcomes. Organization, resources, and communication will be essential to realize this potential.

## SUMMARY RECOMMENDATIONS

1. Create the International COPD Genetics Consortium (ICGC) - to be open worldwide to include all study populations meeting minimum criteria for size, spirometric data, and DNA availability.
2. Mandate of the ICGC is to:
  - Use pooled resources to define rare and common genetic determinants of COPD
  - Identify COPD subtypes and their genetic basis
  - Develop new disease classifications for COPD
  - Foster development of new therapeutic interventions that are subtype or disease classification specific
3. Recommended committee structure:
  - Steering Committee (with oversight of major decisions)
  - Planning/Executive Committee
  - Imaging Committee
  - Phenotype Harmonization Committee
  - Genotyping and Genomics Core
  - Analysis Core
4. Generating new GWAS/genotyping/sequencing/gene expression data
  - Expand and extend existing and ongoing genetic analysis projects
5. Plans for genetic analysis
  - Initial meta-analysis focused on common definitions of case status and on extreme phenotypes
  - Common standardized quality control approaches to clean data
  - Standard approach for data analysis
  - Separate meta-analysis for each racial and ethnic group
  - Replication in study populations not having genome-wide SNP data
6. Data sharing
  - Optimize data sharing while protecting privacy and personal health information
